# Supplementary material for: Colonization times in Moran process on graphs
Source: PLoS Comput Biol. 2025 May 5;21(5):e1012868. doi: 10.1371/journal.pcbi.1012868 (PMC12052132; doi:10.1371/journal.pcbi.1012868)
Supplement: Attachment — Submitted filename: COMMENTS TO THE AUTHOR_.pdf [file pcbi.1012868.s001.pdf]

COMMENTS TO THE AUTHOR:

The topic of this paper is a modified version of the Moran Birth-death process, in which the relative fitness of the mutant is infinite, i.e. only the mutants reproduce and not the residents. The authors have derived tight upper bounds on fixation times for both directed and undirected graphs under this modified Moran process. The main findings are that the authors found an upper bound $O(n^3)$ for fixation time, and they showed via construction that this bound is tight. Additionally, they also found better bounds ($O(n^{2.5})$) for undirected graphs and regular undirected graphs ($O(n^2)$). These bounds are in contrast to the fact that for finite mutant fitness advantage there are spatial structures for which fixation time is exponentially large in the population size n, as well as the $O(n^4)$ upper bound on undirected graphs [35]. Although assuming infinite mutant fitness simplifies the stochastic process somewhat—essentially leading the Markov chain to move in one direction—this "ecological scenario" has received attention in the literature, as noted in the Introduction.

Overall, the paper is a strong addition to the field of evolutionary graph theory. The rigorous proofs, especially those included in the Supplementary Information (SI), are impressive. I recommend publication in PLoS Computational Biology. Below are some specific comments and suggestions.

Comments:

-abstract: 'unique spatial structure' I believe the uniqueness hasn't been proven.

SI:

- Pg 2: (Definition 3) It might be worthwhile to note here that the updating rule has essentially changed from **B**irth-death, to birth-death, because of the random selection for reproduction, while the fixation probability still remains the same.

- Pg 3: (Thm 5 , Proof) I believe the inequality should be $E[t_{Lk}] \geq E[t_k]$ reversed.

- Pg 4: (Thm 6, Proof) Could you double-check if this equality holds true.

$$\frac{1}{|M|} \cdot \frac{1}{\sum_{(u,v)\in E_s} \frac{1}{|M|} \cdot \frac{1}{\deg u}} = \frac{1}{\sum_{(u,v)\in E_s} \frac{1}{n} \cdot \frac{1}{\deg u}},$$

- Pg 5: The proof of Thm 2 is quite neat and elegant !
- Pg 6: (Proof of Thm 4 and Thm 7) I am curious about the motivation behind the factor of ¼ that appears in the proof. For example, the cases $e_k >= ¼ d$ and $e_k < ¼ d$ are considered. Is the factor of ¼ necessary here? Or could we use, say, for instance ⅓ ?

- Pg 7 (Thm 9) What is the relation between G and G_n? Also, if I understand correctly, the family of star graphs with increasing number of nodes would be a non-example of the G_n in question here. It might be a good idea to mention that because otherwise a priori it might seem that all undirected graphs satisfy the constraints of the G in Thm 9.

- Pg 11 (Thm 13 Proof) It should be a+b+1 instead of m+n+1.

- Pg 13 (Thm 15 Proof) The node in the clique that is connected to the line, has one more node than the rest of the nodes in the clique. So, when k=1, the probability of gaining a new mutant would be $\frac{1}{n} \cdot \frac{n-\sqrt{n}-1}{n-\sqrt{n}}$ instead of $\frac{k}{n} \cdot \frac{n-\sqrt{n}-k}{n-\sqrt{n}-1}$. The proof should still go through but will require minor modifications.

Optional (but I am personally interested in the answers!)
- **Temperature/uniform initialization:** As the authors mentioned in the Discussion section, they specified the starting node. Would the authors have like to comment on what they believe the results would look like under different initializations.
- **Amplifiers/Suppressors of selection:** I would be interested in the exploration of any possible connections between colonization times and amplifiers and suppressors of selection. Of course, this would also depend upon the updating rule.
- **Updating rule:** While most papers restrict themselves to a single updating rule, in this instance, as a result of the relative simplicity of the stochastic process, the authors might also be able to extend some of their results to the death-Birth updating rule.
