# Supplementary material for: Colonization times in Moran process on graphs
Source: PLoS Comput Biol. 2025 May 5;21(5):e1012868. doi: 10.1371/journal.pcbi.1012868 (PMC12052132; doi:10.1371/journal.pcbi.1012868)
Supplement: Attachment — Submitted filename: Response to Reviewers.pdf [file pcbi.1012868.s002.pdf]

Response to Reviewers

Reviewer #1: The paper is on evolutionary processes in structured populations that can be represented by graphs. The focus is on the time that is required for mutants' fixation on graphs for two versions of the Moran process in the extreme case where $r \to \infty$. While the paper is well-written and there is nothing wrong with it, the extreme case that is considered by the authors may not be biologically relevant, and hence it may be of less interest. In particular, from an evolutionary point of view, there is actually no competition in the process, resident individuals never reproduce, and mutants inevitably will fixate with probability equal to one given that there is a path from every node to any other node in the graph. The processes in general are oversimplified and some of the results and conclusions are obvious given the nature of the processes that are considered. In many cases, even though valid bounds of the time to fixation are derived, it is not clear that these are tight. The meaning and importance of these bounds is also not clear for a general graph. The approximations on 'specific graphs' (e.g. circle, complete, star graphs) may not be of much interest either – exact solutions of the absorption and fixation times have been derived for every fitness scenario, even for scenarios where the fitness advantage of mutants is not constant. On the other hand, while there is indeed a place for such a study in the literature, I am not sure if this is a good fit for PLOS Computational Biology, as there is nothing 'computational' in the study.
Thank you for your assessment and for constructive suggestions and comments that helped us improve the presentation of our results. Below we answer how we addressed the points.

Some specific comments

Title – I am not sure if the processes that are considered here are evolutionary processes that could be called 'Moran processes'
This is a good point. Since the process we consider is a modification of the Moran process (adapted to the case of infinite r), we prefer to keep the keyword "Moran"

Abstract – there is no mention of 'graphs', but the focus is on 'graphs'. The authors mention 'spatial structures', but this can mean many different things. When they mention 'spatial structures with two-way connections', they may also want to specify that they mean undirected graphs, or explain briefly what they mean by this (also in the 'Author summary' and 'Introduction').
We replaced most occurrences of "spatial structure" with "graph", except for e.g. the Author summary, where we prefer to have the description less "mathematical" (so we use "layouts" rather than "graphs").

Author Summary (page 1):
'We give precise formulas …such as the well-mixed populations, cycles, or stars' – there are exact formulae for these in the literature for every fitness scenario – The authors could potentially refer to these to verify some of their results in the extreme case where $r \to \infty$.
Good point. Generally speaking, our results are indeed in alignment with the literature (as e.g. in refs [20] and [25], see also the 4th paragraph of the introduction). However, we note that the results do not immediately translate, because we consider the modified process instead of the standard Moran process. For example, it is known that on the complete graph

Kn with infinite r the fixation time of the standard Moran process is $N*\log N+o(N*\log N)$, whereas for our modified process it is $2N*\log N + o(N*\log N)$.

‘We prove a tight general bound’ – I have not been convinced that all the bounds presented are tight – but all are indeed bounds of the times considered.
It is true that our upper bound for lattices is not exactly tight, it is only asymptotically tight (because the colonization time on the cycle is $\Theta(n^2)$, as stated just before the Discussion). We toned down the phrase in the author summary to make this clearer.

Introduction

Page 2, ‘migration patterns’ – the authors may want to rephrase, as migration may mean different things, for example real migration of an individual occupying a node, to some other node, leaving the previously occupied node empty.
To help avoid confusion, we now use the phrase “dispersal patterns”.

Page 2, ‘The evolutionary dynamics is governed by the Moran Birth-Death process’ – Many other different processes have also been considered.
This is indeed true. We rephrased the sentence to highlight that the Moran Birth-death process is only one of the options, and we added a short section in the SI where we discuss the death-Birth updating.

Page 2, ‘the number of steps is roughly nlogn’ – is this true for finite populations?
Yes, this is true, provided that the “roughly” hides a constant factor (e.g. 2). In particular, for finite populations and fixed $r>1$, the (conditional) fixation time satisfies

$$T(Kn,r) \approx (N-1)H_{N-1} * (r+1)/(r-1)$$

and the (unconditional) absorption time satisfies

$$AT(Kn,r) \approx (N-1)H_{N-1} * (r+1)/r,$$

where $H_{N-1}=1/1+1/2+1/3+\dots+1/(N-1) \approx \log N$. So both times are of the order of $N\log N$. In particular, for $r=1+s$ and s small we have $T(Kn,r)\approx 2/s * N\log N$ and $AT(Kn,r)\approx 2*N\log N$. We now explain the way we use the word “roughly” in the text.

Page 3, ‘In the opposite limit’ – apart from the two extremes, we also have the more natural case where $r>1$, sometimes it is called as ‘the strong selection case’, where r takes intermediate values. The authors may want to also mention this.
We agree that the regime “$r>1$ fixed” is an important and natural one. Right now, we discuss it in the 4th paragraph of the introduction, before moving on to the special limits $r\to 1$ and infinite r.

Again, the limit of $r\to\infty$, may not describe any natural evolutionary processes.
Indeed, the regime $r\to\infty$ does not involve any competition, since only one type of individuals are reproducing. But we believe that it still makes sense to consider the process, as it could model the setting where individuals of a single type invade and colonize an otherwise empty environment.

Page 3, ‘For every population size n we precisely pinpoint the unique population structure with the slowest colonisation time’ – does the slowest structure depends on the size of the population n?

No, the “structure” of the slowest graph is always the same: for each fixed population size n, the graph with the slowest colonization time among all the graphs on n nodes is the backward graph Bn. (So B100 is the slowest graph among those on 100 nodes, etc.)

Model

Page 3, ‘lattices of any connectivity’ – edit to ‘of any degree’, as here the authors talk about regular graphs.
Done!

Page 4, Colonization time: ‘We will often study the worst-case scenarios’ – the authors should specify what they mean by ‘worst-case scenarios’ – throughout.
The phrase “worst-case” was probably more confusing than helpful, so we removed it (1x in the main text, 1x in the SI).

Results

Page 5, ‘exactly tight’ – what do the authors mean by this?
What we mean is that (for each n) there exists a graph for which the colonization time is exactly equal to the quantity stated in the bound. Maybe this is not proper English? We removed the word “exactly”.

Page 6, ‘colonization times range from roughly…’ – what is ‘roughly’? – throughout
By “roughly” we mean “asymptotically equal to, up to constant multiplicative factors and lower order terms.” We now explain this in the paragraph on asymptotic notation.

Page 6, Under Theorem 3, ‘the colonization times are generally substantially shorter than the fixation times’ – it should be ‘than the fixation times in the case where r>1’, because what the authors call colonization time is eventually the fixation time in the extreme scenario where t->infinity. In addition, I think it is obvious that the ‘colonization times’ will always be lower than that of the the r>1 case (also, ‘Theorem 5’).
This is an important point. We have several comments:
1) Thanks, we reworded the sentence to clarify that we are comparing the colonization time with the fixation time in the case where r>1. (We also clarified that “substantially” means “asymptotically”.)
2) We note that what we call the colonization time T is not quite the fixation time in the scenario r->infinity, because the quantity T is defined for the modified process, rather than for the original process. The quantity that corresponds to the fixation time at r->infinity is the quantity T_L from Theorem 5. As shown there, we have T>T_L (btw we agree that this claim is fairly obvious), so even if T_L was lower than the fixation time, that would not necessarily imply that T was lower too.
3) Moreover, in our opinion it is not obvious that T_L is lower than the fixation time for fixed r>1. For example, it is generally not true that increasing the fitness advantage r decreases the fixation time, see e.g. Fig.2 in https://doi.org/10.1371/journal.pcbi.1012299 and the following discussion.

Page 6, below Theorem 4: ‘Thus, regular graphs’ should be ‘Thus, regular undirected graphs’? ‘than undirected graphs’ –> ‘than other undirected graphs of the same size’?

Thank you, done!

Discussion

Page 8: 'On average, this happens after some number of steps' – edit.
We replaced "some number of steps" with "a certain number of steps" – does that help?

Supplementary

Page 1, Definition 1 – delete 'And' after the first full stop.
We removed the full stop and decapitalized, which hopefully also works.

Page 1, 'u for reproduction is selected proportionally' -> 'u is selected for reproduction proportionally'
Thank you, done!

Page 2, 'a modified version of the classical Moran B-D process' – I am not sure if this version could be called a version of the Moran process.
Thank you for this remark. After discussing it a bit, we prefer to keep calling this process a modified version of the Moran process, since it is equivalent to the Moran process with infinite r, except that the units in which the time is measured are different.

Page 2, 'which captures the idea of a strong selection of mutants' – is this a natural evolutionary process? There is no competition, and mutants are selected randomly.
In the 'Classic Moran process', residents are never chosen 'for reproduction'. What is the biological relevance of this? On the other hand, in the 'Continuous process', residents can be chosen 'for reproduction', but they never reproduce, and the steps in which there are chosen are counted in the fixation time. Apart from the mathematical exercise, it would be good if the authors could clarify the biological/evolutionary process that they model. This is not colonisation of an empty environment either.
Our primary motivation is exactly to model a colonization of an empty environment. There, only the mutants reproduce. However, selecting a mutant for reproduction in every single step would create a bias when measuring time. Intuitively, once there are 10 mutants, the reproduction events should happen roughly 10x more frequently as compared with the case of a single mutant (because the 10 mutants are reproducing "in parallel"). The modified process accounts exactly for this bias and rectifies it. In the section Discussion, we talk at length about why we believe this way of measuring time and defining the modified process makes sense, and we highlight some problematic consequences that occur when one uses the standard Moran process.

Page 2, 'and m spreads into u' -> 'and the offspring of m replaces the individual in u'.
Done!

The authors refer to the second version of the 'Moran process', sometimes as 'continuous', sometimes as 'modified'. It would be good to be consistent. In addition, I would avoid the term 'continuous' as this may imply the continuous-time process.
That's a very good point. We now go with "modified" everywhere.

Page 4, Proof of theorem 6: ‘of gaining a mutant’ -> of gaining an active edge?
We prefer to keep gaining mutants. It is true that a new mutant is gained if and only if the reproduction event happens along an active edge. We reworded it to clarify.

Double check – is the 1/n in the second summation correct?
Thank you, we fixed that!

Again, the process is oversimplified here, and there are no surprises.
We agree that this result is not surprising. We now explicitly say this before stating the result.

Citations in the Supplementary should be ‘numbered’, as in the main part.
Thank you, we fixed that!

Page 4, Section 2.1: ‘is always faster’ - > ‘is always faster than’
Done!

Page 4, Theorem 1/Proof – This is indeed a bound, but not obvious to me that is tight.
At this point, it should not be obvious why it is tight, we clarified that.

Page 5, Theorem 2/Proof: Why this is the slowest?
This is the slowest graph because it matches the expression from the previous general bound. We clarified that.

What is DAGs?
DAGs are directed acyclic graphs (directed graphs that do not contain any cycles), we now say this in the text.

Page 6, Theorem 8/Proof: Not clear to me that this bound is tight? What about the probability of replacement? This is only true in the well-mixed case where the probability of a mutant that is chosen for reproduction replacing a resident is equal to 1 in this particular case. The actual process in each theorem (Classic or modified) should also be specified.
This bound is not tight, just asymptotically tight (because the colonization time on the complete graph is \Theta(n\log n)), we clarified this. The actual process is always specified by the notation we use: T(.) for the modified process, and T_L(.) for the limit process, as described in Preliminaries. Most of our results are on the modified process.

Page 6, ‘of gaining a mutant in stage k’ -> ‘of gaining an active link’ ?
As before, we gain mutants via active links.

Page 6, Theorem 4 – Figure 4 does not show a regular graph.
Good point, we added a comment “some edges not shown” in the figure legend.

Page 6, ‘Or ek is small, but then the new mutant will have many active edges’. Is this still for regular graphs? What about the case of the circle?
When G is the circle, we have ek=2 and d/4=2/4=½. Since ek>½, it is always the first case that occurs (ek is “large enough”).

Last line of Page 7 – this is just the probability of gaining an active link, and not a new mutant, no?
It is both – it is the probability that the reproduction event happens along an active link, and thus it results in us gaining a new mutant (an active link always connects a mutant to a resident).

Page 8, proof of theorem 3: 'the probability that edge (u,v) is selected for reproduction' – edit.
We changed this to "probability that the reproduction event happened along the edge (u,v)."

Page 8, Proof of theorem 9: It is not clear how tight this bound is given the assumptions, but of course Pk>=1/Dn and E[tk]<=Dn.
Indeed, the proof involves several steps, some of which might be non-tight. But the overall bound of T(G_n)=O(n^2) is asymptotically tight, as witnessed by the cycle graph.

Section 5 – I am not sure how much interesting this section may be as long as there are exact formulae for the specific cases presented. The authors could just verify their results with those of previous studies.
We note that the existing results do not immediately translate to our setting, because we consider the modified process and not the the standard Moran process. For example, it is known that on the complete graph Kn with infinite r the fixation time of the standard Moran process is N*logN+o(N*logN), whereas for our modified process it is 2N*logN + o(N*logN).

Page 9, Theorem 10: 'Hence, at every time, exactly two active edges connect the ends' – unless there are n-1 mutants.
The claim is actually true even in this case, the two edges then share an endpoint. We clarified this in the proof.

Clique vs Complete graph – it would be good to be consistent with the use of the name.
That's a good point. We are now using a Complete graph throughout.

Page 10, Section 5.3: 'can be obtained by gluing two copies' ->'can be obtained by connecting the centre of two copies'
Done!

Figure 5 – the circle could also be added here as long as it is considered in this section.
Good idea – we added the cycle graph too.

Page 11, Theorem 12: 'the probability of gaining a new vertex' -> the probability of gaining a new mutant'?
Done!

WLOG – good to write it
We now write it in words.

Theorem 15: This is only on the 'lollipop', no? It should be clarified. Similarly with Theorem 16.

The theorems claim that there exist graphs with some curious properties. Our proof indeed works by demonstrating that the properties are satisfied for the Lollipop graph. However, other graphs might have those properties too. Thus, we prefer to keep the focus away from the particular graph, and onto the fact that for some graphs such curious properties hold.

Reviewer #2: Uploaded review as an attachment

COMMENTS TO THE AUTHOR:

The topic of this paper is a modified version of the Moran Birth-death process, in which the relative fitness of the mutant is infinite, i.e. only the mutants reproduce and not the residents. The authors have derived tight upper bounds on fixation times for both directed and undirected graphs under this modified Moran process. The main findings are that the authors found an upper bound O(n3) for fixation time, and they showed via construction that this bound is tight. Additionally, they also found better bounds (O(n2.5)) for undirected graphs and regular undirected graphs (O(n2)). These bounds are in contrast to the fact that for finite mutant fitness advantage there are spatial structures for which fixation time is exponentially large in the population size n, as well as the O(n4) upper bound on undirected graphs [35]. Although assuming infinite mutant fitness simplifies the stochastic process somewhat—essentially leading the Markov chain to move in one direction—this “ecological scenario” has received attention in the literature, as noted in the Introduction.

Overall, the paper is a strong addition to the field of evolutionary graph theory. The rigorous proofs, especially those included in the Supplementary Information (SI), are impressive. I recommend publication in PLoS Computational Biology. Below are some specific comments and suggestions.
Thank you for the nice words and the many good suggestions below. We took care of all of them and below we specify how we handled them.

Comments:

-abstract: ‘unique spatial structure’ I believe the uniqueness hasn’t been proven.
That’s a good point! We removed the word “unique”. (We are convinced that the structure is unique, but proving it does not seem particularly exciting or enlightening.)

SI:

- Pg 2: (Definition 3) It might be worthwhile to note here that the updating rule has essentially changed from Birth-death, to birth-death, because of the random selection for reproduction, while the fixation probability still remains the same.
The way we think about the modified process, it is exactly the same as the original Birth-death process, except that the time “passes” at a somewhat different rate. So the fixation probability indeed remains the same. In our opinion, it is not quite a birth-death process, because the residents never reproduce (so fitness still plays a major role). We added a brief comment right after Definition 3.

- Pg 3: (Thm 5 , Proof) I believe the inequality should be E[tLk] ≥ E[tk] reversed.
- Pg 4: (Thm 6, Proof) Could you double-check if this equality holds true.
- Pg 5: The proof of Thm 2 is quite neat and elegant !
Thank you, we fixed that!

- Pg 6: (Proof of Thm 4 and Thm 7) I am curious about the motivation behind the factor of ¼ that appears in the proof. For example, the cases ek >= ¼ d and ek < ¼ d are considered. Is the factor of ¼ necessary here? Or could we use, say, for instance ⅓ ?
Good question! The constant ¼ indeed does not really matter here – regardless of the constant chosen, the asymptotics is O(n^2). Thanks to your comment we slightly improved the estimate, so now it gives 6n(n-1) instead of 8n(n-1). Had we used ⅓ instead of ¼, the same argument would go through and in fact would yield the exact same total time of at most 6n(n-1). The optimal constant is roughly 1/(3.41), but we prefer to stick with ¼ to make the computation as simple as possible (but indeed, ⅓ would have worked equally well here).

- Pg 7 (Thm 9) What is the relation between G and Gn? Also, if I understand correctly, the family of star graphs with increasing number of nodes would be a non-example of the Gn in question here. It might be a good idea to mention that because otherwise a priori it might seem that all undirected graphs satisfy the constraints of the G in Thm 9.
Thanks, this was a typo, we now replaced G with Gn. As you write, stars are indeed non-examples. We rephrased the statement of the theorem to make that clearer.

- Pg 11 (Thm 13 Proof) It should be a+b+1 instead of m+n+1.
Thanks, fixed.

- Pg 13 (Thm 15 Proof) The node in the clique that is connected to the line, has one more node than the rest of the nodes in the clique. So, when k=1, the probability of gaining a new mutant would be [..] instead of [..]. The proof should still go through but will require minor modifications.
Indeed, good catch. We modified the graph to match the graph in the main text by making the path part directed. This slightly simplifies the argument and takes care of the two corner cases highlighted. And we added a remark that for the undirected path we would get the same result by essentially the same argument."

Optional (but I am personally interested in the answers!)

- Temperature/uniform initialization: As the authors mentioned in the Discussion section, they specified the starting node. Would the authors have like to comment on what they believe the results would look like under different initializations.
There are two comments that we can make:
First, anytime we prove a bound that holds regardless of the starting node of the mutant, that bound will in fact also hold for all initializations (including uniform and temperature) because any initialization is some sort of a weighted average. We added such a remark to the discussion.
However (and this is the second point), a stronger statement may be true for the average. E.g. it is consistent with our current knowledge that Stars are the slowest undirected graphs under uniform initialization (having time \Theta(n^2\log n) from any starting node), but at the same time for some specific graph G_n and a specific starting node v the time is \Theta(n^{2.5}).

- Amplifiers/Suppressors of selection: I would be interested in the exploration of any possible connections between colonization times and amplifiers and suppressors of selection. Of course, this would also depend upon the updating rule.
We agree, this sounds like an interesting area of future research.

- Updating rule: While most papers restrict themselves to a single updating rule, in this instance, as a result of the relative simplicity of the stochastic process, the authors might also be able to extend some of their results to the death-Birth updating rule.

This is a good point. It turns out that identifying the slowest and fastest graphs under death-Birth updating is substantially simpler. Briefly: We again consider the modified process. This time, in each step we pick a random individual, and if that individual is a resident, it dies and the empty spot gets replaced by a neighbor selected proportionally to fitness (so if the focal individual had at least one mutant neighbor, it becomes a mutant). It is not hard to show that in this case, the slowest graph on N vertices is a path (directed or not) if the initial mutant appears at its end. The corresponding colonization time is N^2-N. In contrast, the shortest possible colonization time occurs if the initial mutant is connected to all other nodes (regardless of how those nodes are connected), and is equal to $N*H_{N-1}=\Theta(N\log N)$. We added those results in the SI and we point to it from the main text.

Reviewer #3: This paper investigates the population graph structure dependence of colonization times in a simple model of evolutionary dynamics (analogous to an infinite fitness Moran model). The authors present new bounds (supported by rigorous proofs in the SI) on mean colonization times for all graph structures, explicitly construct the worst-case graphs with slowest colonization, and refine these bounds for a variety of special cases of interest. They also provide an insightful discussion regarding timescales of the Moran process and identify counterintuitive nonphysical results that can arise if one interprets Moran steps as real time. Given the generality and rigor of the results, this paper will be a valuable contribution to the evolutionary dynamics literature. The concluding discussion regarding notions of time will be particularly useful for interpreting implications of simplified models (like the Moran model considered here) for real world evolutionary processes. Below I detail several minor clarifying questions about the methods and results; once these are addressed I will fully support publication of this paper.
Thank you for encouraging words and for good questions. We answer the below.

1. The lower bound shown in Figs. 2,3 should be briefly described in the main text. I would naively expect the complete graph would have fastest colonization, is it obvious whether or not this is true?
The lower bound is stated in a paragraph just after Theorem 2 (the full statement and proof appears in the SI). We now also added this to the figure legend. About the second question: It is not obvious to us. Also in the setting of a fixation time (rather than a colonization time), we are not aware of a result that would state that the complete graph is absolutely the fastest one – but same as here it is known to be asymptotically optimal (i.e. taking of the order of n\log n steps)

2. How would the authors modify the standard Moran update rule for the "continuous process" when the fitness ratio is finite? Can the initialization comparison in Fig. 4 be made for the continuous update rule as well? Is the modified process monotone (in the sense of SI section 6.2)?
We answer the 3 questions separately:
1) One way to do this would be as follows: Sample steps of the standard Moran process, and together with each step, define the amount of time that "passes" as 1/F, where F is the total fitness of the population. So for example on a complete graph Kn with k mutants, the amount of time that passes would not be 1/n but 1/(k*r + (n-k)*1) – note that this would depend on k. Admittedly, this is not as clean as the case of infinite r.
2) Yes, the figure showing the comparison is below.

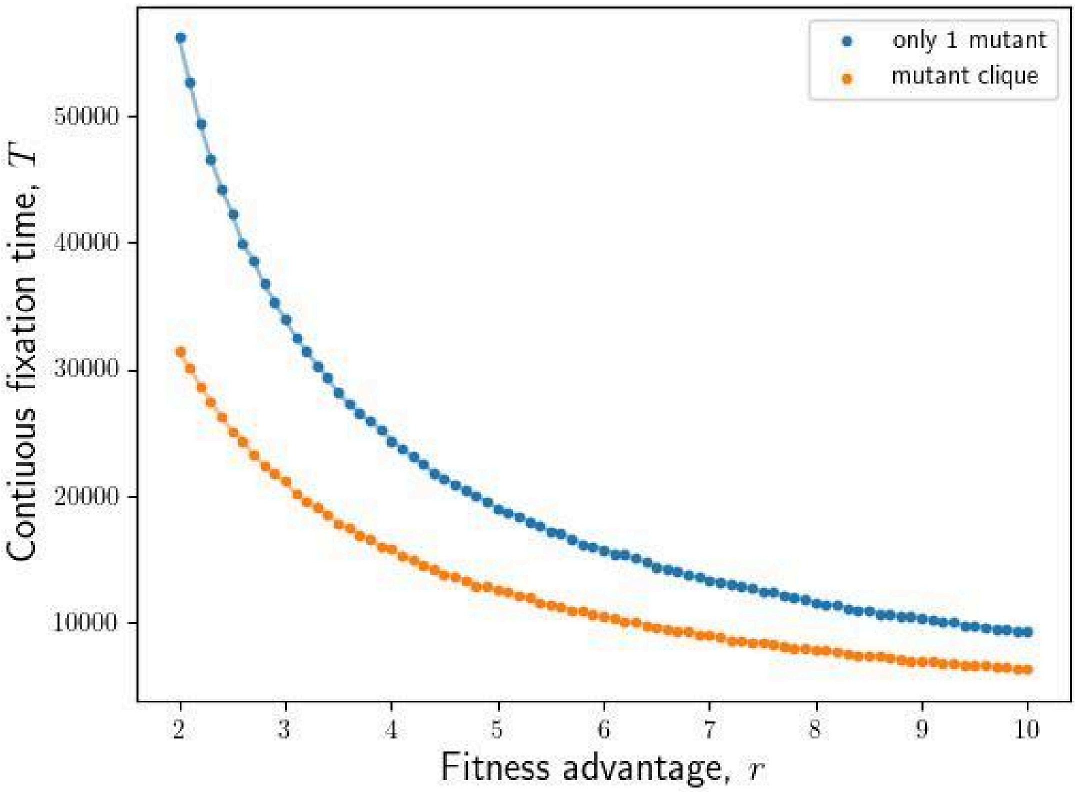

3) We believe that it *is* monotone, using a standard coupling argument: just very briefly, if we compare 2 worlds where mutants occupy sets S\subset S' of nodes, then after one step the resulting new states {S1,S2,..,Sk} and {S1',S2',..,Sk'} of mutants can be coupled such that the invariant Si\subset Si' is preserved. (Since the second process always has a super-set of mutants, it can not finish later than the first one.)

3. Do the authors expect the modification of the Moran update rule used in this paper to impact any well-known results in the literature? For example, the authors mention (line 26) the fact that amplifier graphs that increase fixation probability always increase fixation time, is it clear whether this is still true for the modified process?
First, the statement about an increase in fixation time only applies to so-called strong amplifiers (aka "superamplifiers"). As far as we can tell, there could be (weak) amplifiers that are as fast as the complete graph (in terms of the standard update rule). We do not dare to conjecture what happens for the modified update rule.

4. Since this paper focuses only on means, it would be interesting to slightly expand upon the statement (line 224-5) regarding the concentration around the mean fixation time. Besides a few special cases covered in the cited papers, it is not clear to me that the distribution of fixation times is generally well concentrated, particularly for directed graphs. Could the techniques developed in this manuscript be used/generalized to bound higher order moments? What would be the evolutionary implications of large variation in fixation times?
Those are interesting and difficult questions. We can make one comment about the concentration: In general, for any graph (directed or not) an upper bound B on the expected

time automatically implies that with probability 1-1/n the process terminates in at most \Theta(B*log n) steps, so it is relatively well concentrated. The argument goes by considering epochs of length 2*B. Within each epoch, the process terminates with probability at least ½ (by Markov inequality). So within the first log n epochs it terminates with probability 1-(½)^log n = 1-1/n.

5. For theorem 3 in the SI it would be helpful to better motivate and explain the choice d=sqrt(n). E.g. how does the proof breakdown if another d is chosen to try to tighten the bound? It seems like the choice balances the time contributions from colonizing neighbors of “small” and “large” vertices; is that the correct interpretation?

This is indeed the case. The proof distinguishes two cases depending on whether there are many (roughly >d) or few (roughly <d) active edges. Ignoring some details, we argue that in the first case, the next stage takes at most roughly n^2/d steps, and in the second case it takes at most roughly n*d steps. Equating, we get d=\sqrt{n}. For d<<sqrt(n) case 1 would result in long stages, and for d>>sqrt(n) case 2 would result in long stages, so other choices of d do not lead to an immediate improvement in the bound.

Minor comments:

— The half path graph, whose colonization time is plotted in Fig. 2 is not mentioned in the main text.

Good catch, we briefly explain it in the figure legend, pointing to SI for a full definition.

— SI Theorem 5: Should the inequality involving expectation of t_k, t_k^L be reversed?

Thank you, we fixed that…

— SI before Definition 9: acronym DAG should be defined.

…and we fixed this too.
